# In need of age‐appropriate cardiac models: Impact of cell age on extracellular matrix therapy outcomes

**DOI:** 10.1111/acel.13966

**Published:** 2023-10-06

**Authors:** S. Gulberk Ozcebe, Pinar Zorlutuna

**Affiliations:** ^1^ Bioengineering Graduate Program University of Notre Dame Notre Dame Indiana USA; ^2^ Department of Aerospace and Mechanical Engineering University of Notre Dame Notre Dame Indiana USA; ^3^ Harper Cancer Research Institute University of Notre Dame Notre Dame Indiana USA

**Keywords:** aging, cardiac declines with age, cellular aging, cytokines, extracellular matrix therapies, gene expression, human heart, human iPSC‐derived cardiomyocytes

## Abstract

Aging is the main risk factor for cardiovascular disease (CVD). As the world's population ages rapidly and CVD rates rise, there is a growing need for physiologically relevant models of aging hearts to better understand cardiac aging. Translational research relies heavily on young animal models; however, these models correspond to early ages in human life, therefore cannot fully capture the pathophysiology of age‐related CVD. Here, we first investigated the transcriptomic and proteomic changes that occur with human cardiac aging. We then chronologically aged human induced pluripotent stem cell‐derived cardiomyocytes (iCMs) and showed that 14‐month‐old iCMs exhibited a similar aging profile to the human CMs and recapitulated age‐related disease hallmarks. Using aged iCMs, we studied the effect of cell age on the young extracellular matrix (ECM) therapy, an emerging approach for myocardial infarction (MI) treatment and prevention. Young ECM decreased oxidative stress, improved survival, and post‐MI beating in aged iCMs. In the absence of stress, young ECM improved beating and reversed aging‐associated expressions in 3‐month‐old iCMs while causing the opposite effect on 14‐month‐old iCMs. The same young ECM treatment surprisingly increased SASP and impaired beating in advanced aged iCMs. Overall, we showed that young ECM therapy had a positive effect on post‐MI recovery; however, cell age was determinant in the treatment outcomes without any stress conditions. Therefore, “one‐size‐fits‐all” approaches to ECM treatments fail, and cardiac tissue engineered models with age‐matched human iCMs are valuable in translational basic research for determining the appropriate treatment, particularly for the elderly.

## Graphical abstract

Our results clearly demonstrated that the advanced aged iCMs, representing the elderly, did not benefit equally from the post‐MI young ECM treatment as the younger iCMs, and were even adversely affected in the absence of stress conditions. There is a need for age‐appropriate cardiac models in translational research to develop personalized treatments for the elderly population, and to move beyond the “one‐size‐fits‐all” approach in ECM therapies.

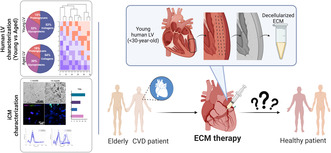

## 1 INTRODUCTION

Age is a significant risk factor for cardiovascular diseases (CVD), including myocardial infarction (MI). Studies show that more than half of CVD morbidity and long‐term mortality following MI occur in individuals aged 65 years and older (Kochar et al., 2018; Virani et al., 2020). Age‐related changes at the cellular, extracellular, and tissue levels negatively impact disease diagnosis as well as therapeutic outcomes. Pharmacological treatment outcomes were reported to be inconsistent and unpredictable for the elderly (Berliner & Bauersachs, 2018). Similarly, despite the demonstrated benefits of cell‐based therapies for MI in preclinical studies, early clinical trials resulted in limited improvement in left ventricular ejection fraction and ventricular remodeling, particularly for the elderly (Stamm et al., 2008). This is mainly due to decreased responsiveness of aged cells to their environment, and consequently to treatments (Ozcebe et al., 2021; Sheydina et al., 2011). Understanding cardiac aging and the effect that this has on CVD therapy outcomes are essential to ultimately prevent and treat age‐associated disease syndromes.

Decellularized extracellular matrix (ECM) is a promising biomaterial for the regeneration and repair of musculoskeletal (Kim et al., 2018), neural (Ren et al., 2018), liver (Shimoda et al., 2019), and cardiovascular systems (Bejleri & Davis, 2019). Studies have reported regenerative capabilities to be more effective when ECM was obtained from young tissues (Ozcebe et al., 2021; Williams et al., 2014). We previously showed the differences in the human induced pluripotent stem cell (iPSC)‐derived cardiomyocytes (iCM) response to young, adult, and aged cardiac ECM. We showed that young ECM increases cell proliferation and drug responsiveness, improves cardiac function overall, initiates cell cycle reentry, and mitigates oxidative stress damage in quiescent state‐aged iCMs (Ozcebe et al., 2021). Moreover, regardless of the ECM age, other groups have demonstrated the feasibility of using ECM for post‐MI ventricular remodeling and cardiac functional recovery (i.e., LVEF) in animal models. Porcine cardiac ECM‐derived hydrogels have been reported to increase the number of endogenous cardiomyocytes while preserving post‐MI cardiac function (Singelyn et al., 2012). Neonatal mouse cardiac ECM was shown to be more effective to prevent post‐MI adverse ventricular remodeling, such as fibrosis, compared to adult ECM (Wang et al., 2019). In another study, zebrafish heart ECM, which is known to be highly regenerative, was reported to exert pro‐proliferative effects and contribute to post‐MI cardiac regeneration in adult mice (Chen et al., 2016). A recent clinical study showed that hydrogels derived from decellularized porcine myocardium improved left ventricular function in post‐MI patients (57–62 years old) (Traverse et al., 2019). Taken together, these studies provided valuable information on the safety, feasibility, and efficacy of ECM as a regenerative and post‐MI therapy. There is a growing trend toward using ECM therapies, alone or in combination with cells, for MI. However, because current studies are largely based on young cells and young animal models, we still have a limited understanding of how the aged heart would respond to ECM therapies. As MI disproportionately affects the elderly, and the therapy outcomes vary with the patient age, here we generated an age‐appropriate heart tissue model using aged iCMs and investigated ECM therapies for both MI treatment and prevention.

In this study, we explored age‐related changes in the human heart. We characterized young (<30 years‐old) and aged (>50‐years‐old) nonfailing human left ventricle (LV) samples and identified genes and proteins strongly altered with aging. We detected higher collagen in aged LV and higher glycoprotein ratio in young LVs. We then compared the aging profile, gene and protein expressions of 3‐month‐old, 6‐month‐old, and 14‐month‐old (advanced aged) iCMs to each other and also to native heart CMs. Our results revealed a high degree of similarity between the advanced aged iCMs and human cardiac aging, in terms of their stress and contractile function impairment‐related transcriptional signatures.

Next, we used chronologically aged iCMs to explore age‐related changes in vitro. We investigated the effects of cell age on ECM therapy outcomes. Our findings showed that ECM treatment outcomes were influenced not only by cell age but also by the presence of stress conditions such as MI. Following MI‐mimicking stress conditions (i.e., hypoxia), young ECM treatment led to functional recovery at all cell ages, with increased survival observed only in 3‐month‐old iCMs.

In the absence of stress, the beneficial effects of young ECM treatment were limited to the younger, 3‐month‐old iCM group. ECM upregulated cardiac structural and functional genes, increased beating frequency and velocity, regulated action‐potentials, and suppressed stress‐related genes and the senescence‐associated secretory phenotype (SASP) in 3‐month‐old iCMs. Surprisingly, young ECM was pro‐aging and disrupted the beating of advanced aged iCMs, caused twitching. These results challenged the widely held assumption of the universal benefit of young ECM, raising uncertainties about the safety and efficacy of employing young ECM as a preventative treatment for CVD in the elderly.

In conclusion, here we reported age‐dependent transcriptional alterations in nonfailing human heart LVs from both young and aged subjects, and in chronologically aged iCMs. To the best of our knowledge, this is the only study displaying transcriptional alterations in human LV with a focus on aging without any disease conditions. Furthermore, our results showed that chronologically aged iCMs are excellent candidates to mimic aged heart behavior and can be used to conduct CVD studies for the elderly. Using an age‐appropriate cardiac model, we showed that the “one‐size‐fits‐all” ECM treatment approach is doomed to fail, as results are highly dependent on cell age and stress conditions.

## 2 RESULTS

### 2.1 Aging‐associated changes in human heart left ventricles

**FIGURE 1:**
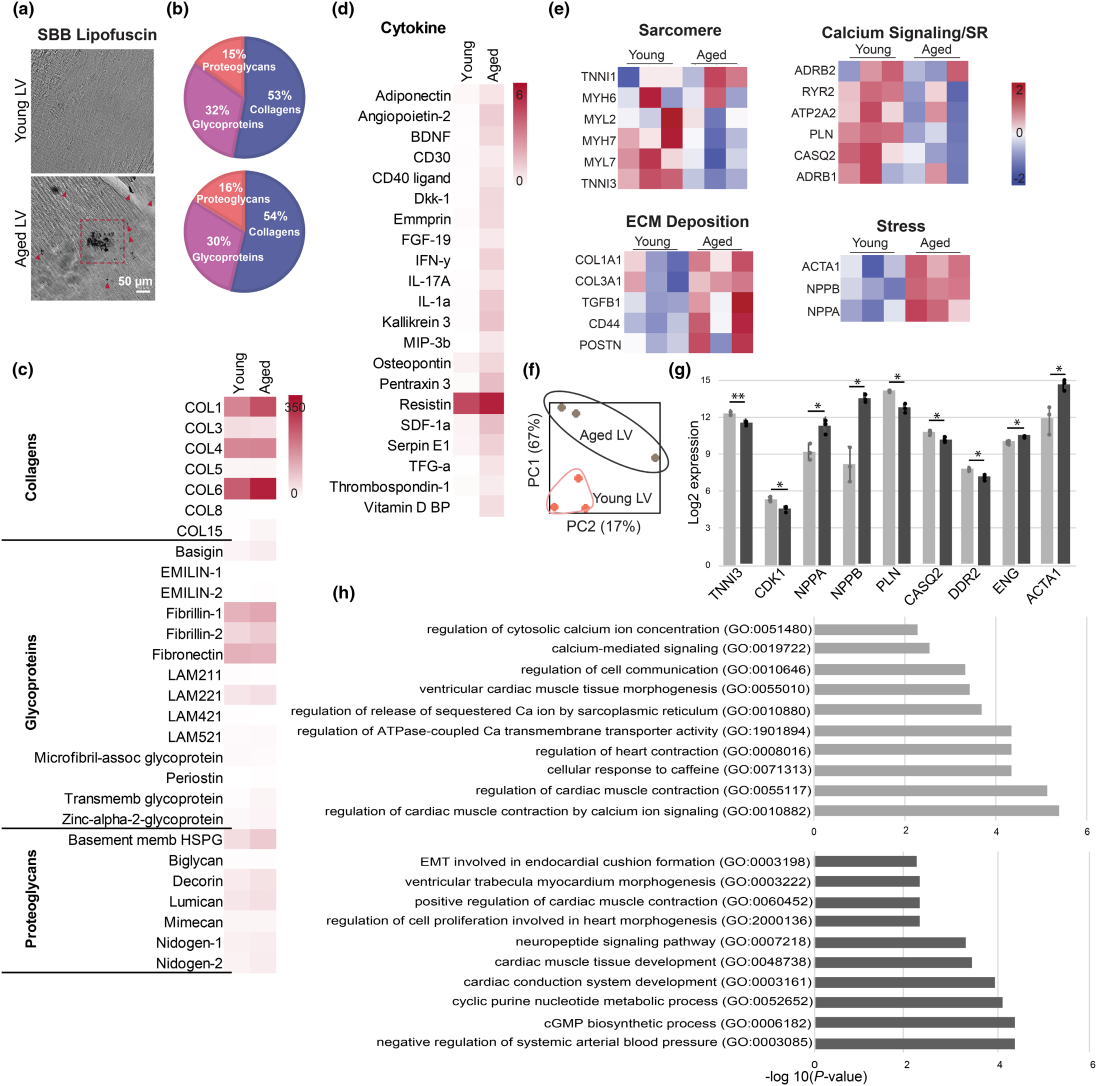
Human heart left ventricle age dependent alterations (a) Sudan Black B staining showing lipofuscin accumulation. (b) ECM compositions of young (*n* = 3) and aged (*n* = 3) human left ventricles (LV) represented as the relative percentage detected by mass spectroscopy. (c) The breakdown of the three main ECM components. Values represent the peak area reflecting ionic species abundance with the respective m/z ratio. (d) Most abundant cytokines detected in human LVs relative to the internal positive control. (e) Heatmaps showing the z‐scores of the key genes involved in distinct features of CM behavior: sarcomere, calcium (Ca^2+^) cycling and sarcoplasmic reticulum (SR), ECM deposition, and stress response. (f) Principal component analysis (PCA) of the gene expression data, depicting the group relationships. (g) Statistically altered gene expressions of young and aged human LV. Statistical analysis was done using one‐way ANOVA with post hoc Tukey's test. **p < 0.01, *p < 0.05, *n* ≥ 3. Data presented as mean ± standard deviation (SD). (h) Gene Ontology analysis showing the biological processes associated with the genes overexpressed in young and aged LVs.

Left ventricles derived from healthy human hearts (young: <30‐years‐old, *n* = 3 and aged: >50‐years‐old, *n* = 3) were characterized. The presence of lipofuscin, a well‐established aging marker, was assessed to validate the age ranges selected for the young and aged LVs. Lipofuscin accumulation was observed solely in the aged LVs (Figure 1a). Mass spectroscopy results showed higher collagen and fibrillin ratio in aged LV, and higher fibronectin and glycoprotein ratio in young LV (Figure 1b,c). In mice, decreasing glycoproteome was linked with age‐related functional decline of the CV system (Franzka et al., 2021), and these results are in alignment with human cardiac aging. Among the less abundant ECM proteins, decorin and lumican which modulate fibril structure and collagen fibrillation were higher in aged LV. These proteoglycans are known to cause thicker collagen fibrils, resulting in lower fibril area fraction, hence poor mechanical properties and functional decline with increasing aging (Figure 1c). Cytokine profiling via dot blot‐based immunoassay also revealed differential concentrations of cytokines present in young and aged LVs. CVD biomarker pentraxin 3 (PTX3) and interestingly kallikrein 3 (KLK3), which protects against fibrosis were more than doubled in aged LVs. Other abundant cytokines detected in aged LV include resistin, IL‐1α, IFN‐γ, angiopoietin‐2, Dkk‐1, Emmprin, SDF‐1α, and SerpinE1 among others (Figure 1d). Proteomapping of the affected KEGG pathways showed upregulation predominantly in NF‐KB, MAPK, and HIF‐1α signaling in aged LVs with essential roles in senescence‐associated secretory phenotype (SASP)‐regulation, resistance to apoptosis and aging‐related pathology, respectively.

mRNA levels related to CM maturity, function, and apoptosis were quantified (Figure S1a) and a 67% variance was detected between aged and young LV samples (Figure 1f). Adult‐type sarcomeric genes (*TNNI3*, *MYH7)* and multiple Ca^2+^ cycling/SR genes were highly expressed in young LV. Myocardial fibrillar collagens (*COL1A1*, *COL3A1*) along with other adverse cardiac remodeling contributors and stress‐related genes were highly expressed in aged LV (Figure 1e). Among screened genes, we identified nine that were significantly altered (*p* < 0.05) by human cardiac aging. Specifically, NPPB, a ventricular natriuretic peptide known to be secreted in the myocardium upon stress, was upregulated 40‐fold (*p* = 0.017) in the aged LV (Figure 1g). The KEGG pathway analysis revealed that the differentially expressed genes (DEGs) in young LV were associated with cardiac muscle contraction, calcium signaling, and focal adhesion pathways, while in aged LV they were associated with HIF‐1, PI3K‐Akt signaling pathways, hence cardiovascular aging, and cardiac disorders. The results of gene ontology (GO) analysis also showed that DEGs in young LV were significantly enriched in biological processes, including the regulation of cardiac muscle contraction by calcium ion signaling, and cell communication. Aged LV DEGs were enriched in cGMP signaling pathways, cardiac muscle tissue development, and neuropeptide signaling pathways (Figure 1h).

### 2.2 Age‐associated changes in chronologically aged iCMs

**FIGURE 2:**
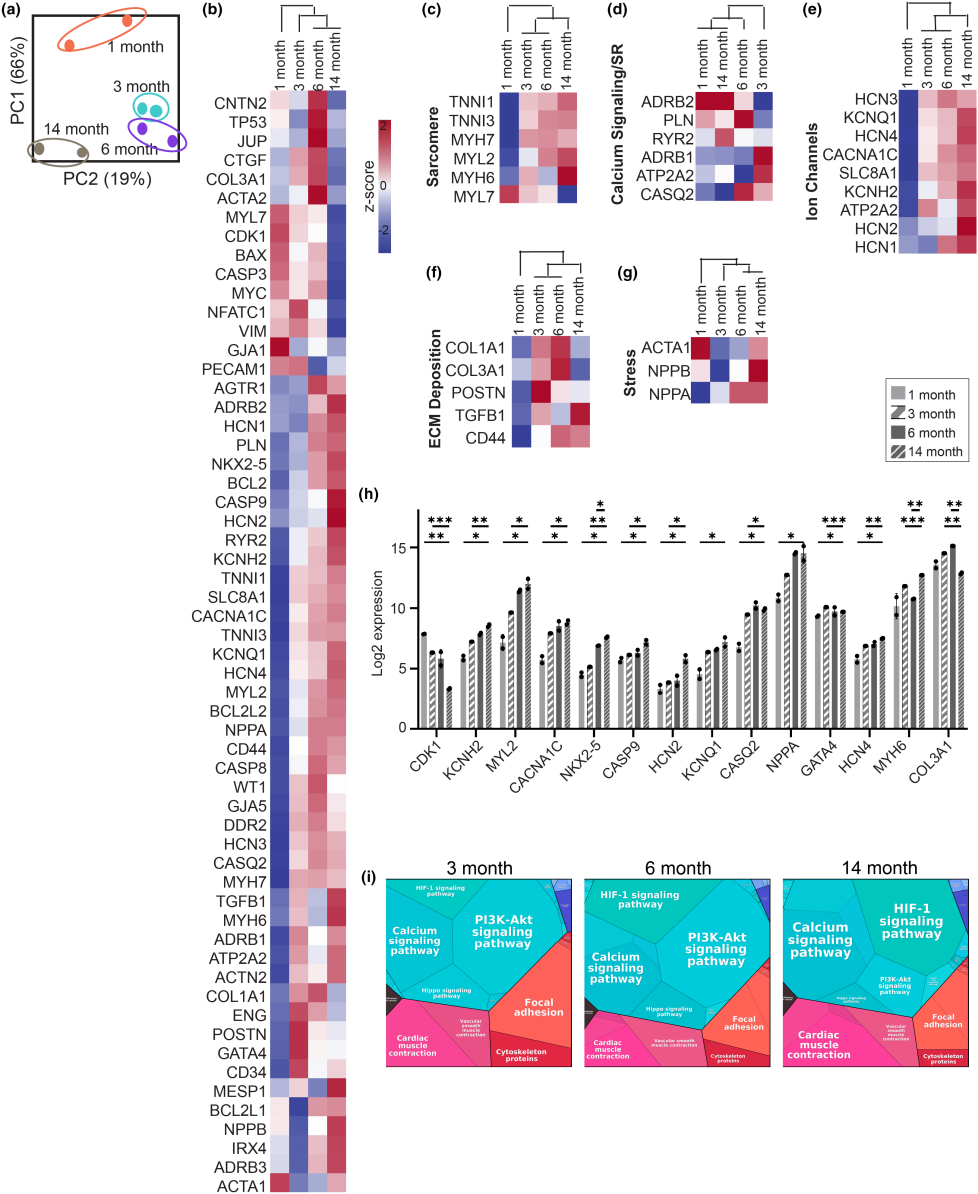
iCM age‐dependent transcriptional alterations (a) Principal component analysis (PCA) of the gene expression data, depicting the group relationships of young and aged iCMs. (b) Differential expression levels of the preselected 58 cardiac and aging specific genes for iCMs. (c–g) Heatmaps of key genes involved in distinct features of CM behavior: (c) sarcomere, (d) calcium (Ca^2+^) cycling and sarcoplasmic reticulum (SR), (e) ion channels, (f) ECM deposition, and (g) stress response. (h) Statistically altered gene expressions of aged iCMs. Statistical analysis was done using one‐way ANOVA with post hoc Tukey's test. *** < 0.001, **p < 0.01, *p < 0.05, *n* = 6 pooled into two technical replicates. Data presented as mean ± standard deviation (SD). (i) Proteomaps showing the KEGG pathways.

Unsupervised hierarchical clustering revealed a 66% variance between young and aged iCMs (Figure 2a,b). GO analysis revealed that genes downregulated in advanced iCMs were significantly enriched in biological processes, including DNA damage response, apoptotic processes, and cell cycle progression. The genes that were upregulated with iCM aging were associated with cardiac contraction, ion transport, and calcium signaling (Figure S1b), indicating acquired structural and functional maturity with prolonged culture time. With prolonged culture, the adult‐type sarcomeric myosins and cardiac troponin were differentially expressed (Figure 2c,h). The intermediate filament protein (*VIM*) that is highly expressed in fetal CMs was downregulated with cellular aging. Although structurally more mature, advanced aged iCMs grouped together with young 1‐month‐old iCMs regarding their calcium signaling and sarcomeric reticulum‐related expressions (Figure 2d). Reduced excitation‐contraction coupling expressions were observed in both immature 1‐month‐old and advanced aged iCMs, indicating that CMs reverted toward an impaired calcium handling machinery as they age. The calcium‐handling genes that are essential for cardiac action potential and cardiac contraction were upregulated up to eightfold in 3‐ and 6‐month‐old iCMs, while the negative calcium import regulator (*PLN*) was upregulated in 6‐ and 14‐month‐old iCMs. Consequently, *PLN:ATP2A2* ratio, an indicator of reduced SERCA activity and impaired calcium handling, was more than doubled in 6‐ and 14‐month‐old iCMs (Figure S1d). Potassium and sodium channel gene expressions were gradually increased with prolonged culture (Figure 2e,h).

Adverse cardiac remodeling genes that mediate cardiac fibrosis were upregulated with iCM aging (Figure 2f). In alignment with the previous single‐cell RNA‐seq data, we observed upregulated expression of collagens and ECM deposition genes in 3‐ and 6‐month‐old iCMs. However, these genes were downregulated in advanced aged iCM. These cells also expressed high levels of cardiac hypertrophy and stress‐related (*NPPA*, *NPPB)* genes. Especially *NPPA* was upregulated more than 16‐fold from 1‐month to 14‐month of culture (Figure 2g). Although experiencing more stress, and significantly upregulated initiator caspase *CASP9* expression (Figure 2h), a predictive value that determines the susceptibility to the apoptotic signal, *BAX/BCL2* ratio revealed that advanced aged cells were two times more resistant to apoptosis at the gene level (Figure S1c).

Additionally, cell‐cycle‐associated *CDK‐1* gene expression was significantly downregulated while *GATA4*, *a* critical regulator of cardiac regeneration, and its cofactor, *NKX2‐5* expressions were upregulated in advanced aged iCM (Figure 2h). The KEGG pathway analysis of the relative expression data further showed that regeneration mediator Hippo signaling and PI3K‐Akt pathways were downregulated, while HIF‐1 signaling pathway and cardiac muscle contraction were upregulated in advanced aged iCMs (Figure 2i).

**FIGURE 3:**
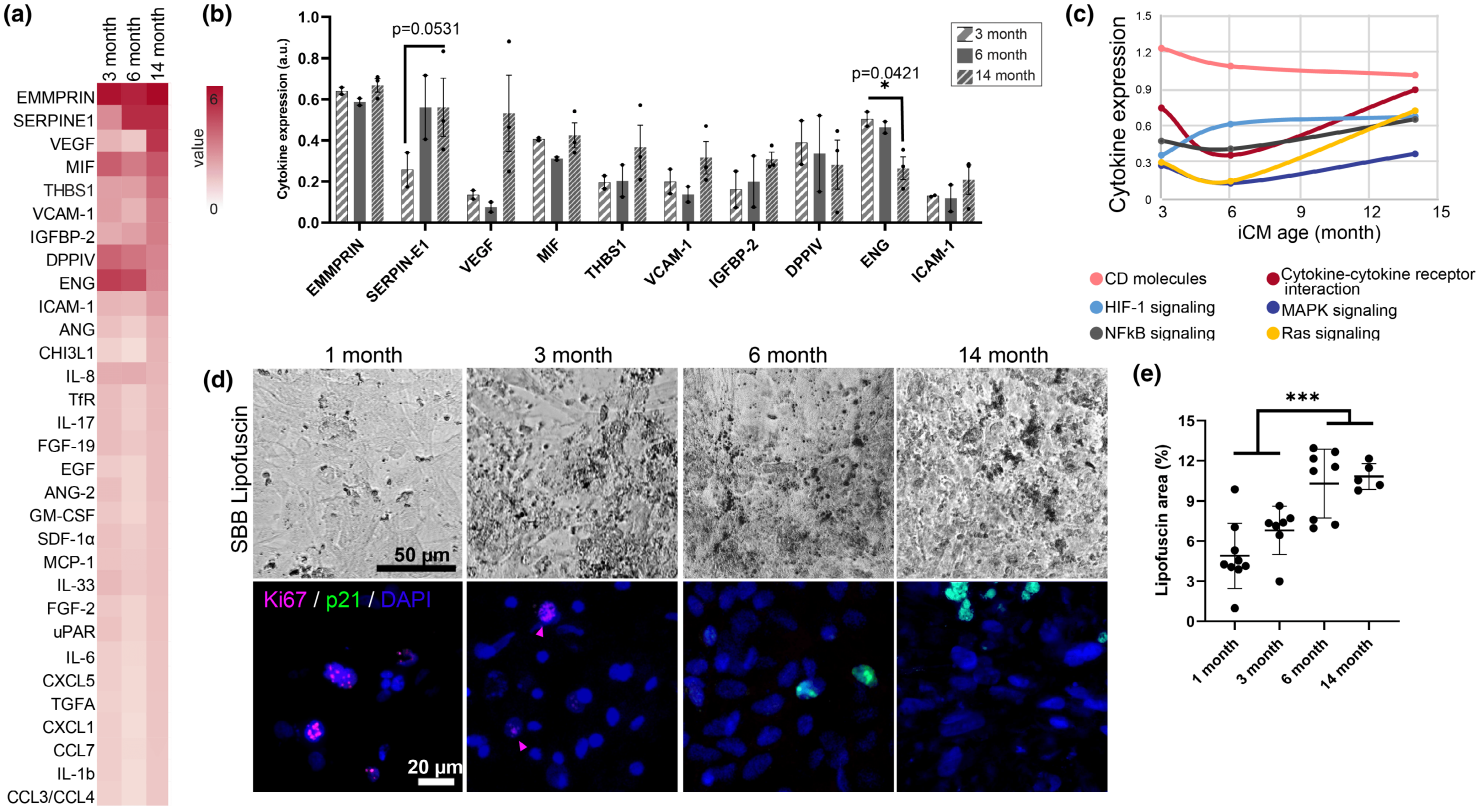
Aged iCM protein and cell level alterations with aging (a) Heatmap showing the screened protein expressions of aged iCMs (b) Highly expressed cytokine expressions of aged iCMs. Statistical analysis was done using one‐way ANOVA with post hoc Tukey's test. ***p < 0.001, **p < 0.01, *p < 0.05, *n* = 6 pooled into ≥2 technical replicates. Data presented as mean ± standard deviation (SD). (c) iCM age dependent changes in the cytokine expressions with respect to their role or involved pathways. (d) Senescence and proliferation‐related staining of the iCMs. The senescence markers lipofuscin (black, top panel) and p21 (green); proliferation marker Ki67 (magenta). Nuclei were counterstained with DAPI (blue). (e) Senescence‐associated lipofuscin accumulation across the iCM ages calculated as the covered area.

Advanced aged iCMs had a larger proteome body, which might indicate increased age‐associated inflammation or inflammageing (Figure 3a). Among the highly detected proteins, the senescence mediator (SERPINE1) increased whereas the critical mediator of cardiovascular health (ENG) decreased gradually with cellular aging (Figure 3b). In agreement with the transcriptomic alterations, the pro‐aging Ras signaling pathway, master senescence associated secretory phenotype (SASP)‐regulator NF‐KB signaling pathway, HIF1α and cytokine‐cytokine receptor interaction proteins that are known to be associated with age‐related degenerations in multiple organ systems (Rea et al., 2018; Shahrokhi et al., 2018) were highly expressed in advanced aged iCMs (Figure 3c). Aged iCMs were further characterized at the cell level via aging and proliferation marker expressions. Lipofuscin accumulation was significantly increased with the increasing iCM age (*p* < 0.001) (Figure 3d,e). Similarly, age‐associated p21 expression was observed at 6‐ and 14‐month‐old iCMs whereas 1‐ and 3‐month‐old iCMs presented Ki67. These results displayed the dynamic changes occur with iCM aging.

### 2.3 ECM treatment effect on post‐MI functional recovery

**FIGURE 4:**
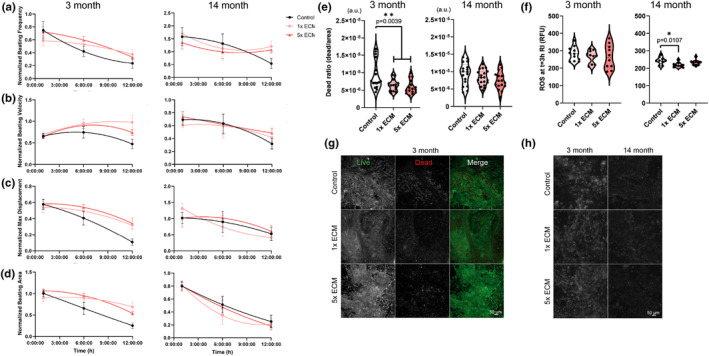
Post MI analysis. Temporal changes of (a) beating frequency, (b) beating velocity of spontaneous cell beating, (c) maximum displacement of a pixel in a frame due to spontaneous beating, and (d) beat area (%) recorded for 12 h post‐MI. Left panel:3‐month‐old, right panel:Advanced aged iCM. Post‐MI (e) cell death ratio and (f) cellular ROS levels of 3‐month‐old 14‐month‐old advanced aged iCMs at 12 h RI. Representative images of (g) 3‐month‐old iCM live dead staining and (h) ROS generation measurement of 3‐month‐old and advanced aged iCM at 3 h RI. Statistical analysis was done using one‐way ANOVA with post hoc Tukey's test. **p < 0.01, *p < 0.05, *n* ≥ 3. Beating recovery was normalized to the pre‐MI initial values and data presented as mean ± standard deviation (SD).

To determine whether there were any cell age‐dependent responses to the young ECM treatment for post‐MI recovery, we exposed 3‐month‐old and advanced aged iCMs to MI‐like stress conditions. The spontaneous beating of the cells was recorded before anoxia, and at 1 h, 6 h, and 12 h RI to assess the beating recovery, and beating frequency (Figure 4a), beating velocity (Figure 4b), maximum displacement (Figure 4c), and beating area (Figure 4d). Three‐month‐old iCMs had higher initial beating frequencies (0.59 ± 0.04 Hz) than the advanced aged iCMs (0.22 ± 0.02 Hz), hence beating values were normalized separately.

Within the first hour post‐anoxia, 3‐month‐iCMs slowed down while advanced aged iCMs displayed rapid, irregular twitches resulting in doubled beating frequencies. At the end of 12 h, 1‐out‐of‐4 samples of 3‐month‐old, and 4‐out‐of‐10 samples of advanced aged iCMs stopped beating. We observed beneficial effects of ECM treatment in both 3‐month and advanced aged iCMs. Control untreated 3‐month‐old group beating frequency, robustness, and area recovered to only half of their original pre‐MI values, while ECM treated groups had faster, stronger beating across a larger area at the end of 12 h normoxia (Figure 4a–d). For advanced aged iCMs, ECM treatment sustained their beating frequency (Figure 4a), and the control group frequency decreased to half of the original value. However, ECM treatment did not affect the beating robustness or area. Regardless of the ECM, the beating area of advanced aged iCMs was dramatically reduced.

### 2.4 ECM treatment effect on the deleterious effects of MI

We investigated the effect of cell age on the therapeutic potential of the young ECM. Regardless of the dose used, ECM significantly lowered the dead cell count of the 3‐month‐old cells (*p* = 0.0039) while the detected cellular ROS levels were comparable (Figure 4e–h). There was no survival difference in the advanced aged iCMs (Figure 4e); however, mitochondrial ROS generation significantly decreased (*p* = 0.0107) with the ECM supplementation (Figure 4f). Advanced aged cells were further screened for apoptosis‐related proteins to investigate why we detected ECM effects on ROS generation but not on cell survival. When relative expressions were compared, we detected high levels of clusterin, cell protectant protein against ROS‐induced apoptosis, ROS scavenger catalase, and low levels of stress proteins HSP60 and HSP70 in ECM supplemented cells. The pro‐apoptotic proteins did not show a difference, yet one of the critical early mediators of apoptosis, Ser46 phosphorylation of p53 level, and the apoptosis initiator, cytochrome C gradually decreased with increasing ECM dose (Figure S2a).

### 2.5 ECM treatment effect on aged iCM expressions without any stress conditions

**FIGURE 5:**
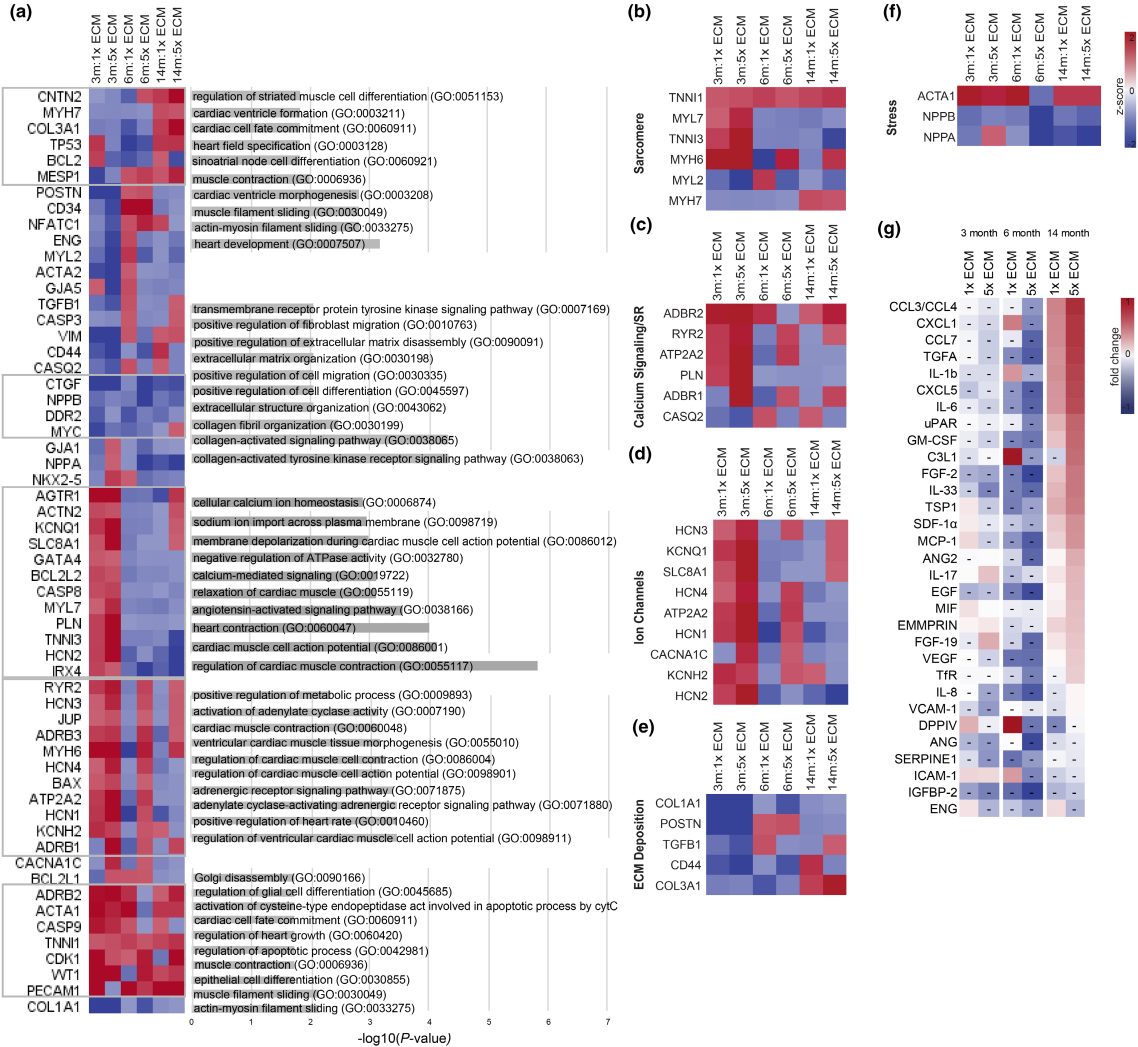
ECM treatment effect on aged iCMs. (a) Differential expression levels of the preselected 58 cardiac and aging specific genes for iCMs. Gene ontology analysis showing the biological processes associated with the upregulated and downregulated genes with iCM aging. (b–f) Heatmaps of key genes involved in distinct features of CM behavior: (b) sarcomere, (c) calcium (Ca^2+^) cycling and sarcoplasmic reticulum (SR), (d) ion channels, (e) ECM deposition, and (f) stress response. (g) Heatmap showing the screened protein expressions as a fold change from their corresponding control groups. *n* ≥ 3.

Following a 10‐day treatment with young human heart LV‐derived ECM at two concentrations (1x ECM: 0.1 mg/mL and 5x ECM: 0.5 mg/mL), we investigated the changes in transcriptome and cytokine levels. Regardless of the cell age or the ECM concentration, the treatment downregulated genes that are associated with collagen activated signaling pathways, extracellular organization, and noncardiac cell migration, and upregulated genes associated with cardiac cell fate commitment and muscle contraction (Figure 5a). GO analysis also showed that genes upregulated upon ECM treatment were enriched in regulation of glial cell differentiation, which we recently reported to be regulators of heart rate (Kikel‐Coury et al., 2021).

Expectedly, high concentration ECM had a greater effect on the cells, and the ECM treatment outcome depended heavily on the cell age. GO results showed that DEGs in 3‐month‐old iCMs were associated with functional processes such as the movement of ions and cardiac muscle contraction and relaxation. Relatedly, sarcomere, calcium signaling, and ion channel expressions were upregulated in an ECM dose dependent manner in 3‐month‐old iCMs (Figure 5b–d). DEGs in 3‐month‐old and high‐dose ECM treated 6‐ and 14‐month‐old iCMs were involved in the positive regulation of metabolic processes in addition to cardiac contraction, and regulation of cell action potential. Adrenergic receptor (*ADRB1*) and ryanodine receptor (*RYR2*) levels which play an integral roles in excitation‐contraction coupling and cardiac energy metabolism (Figure 5c), increased with high concentration ECM treatment. HCN channel family, Ca^2+^ and K^+^ channel levels in 6‐month‐old iCMs, and Na^+^/Ca^2+^ exchanger (*SLC8A1*) levels in advanced aged iCMs increased only when treated with high concentration ECM. Surprisingly, DEGs in 14‐month‐old iCMs treated with ECM were associated with heart development and cardiac ventricle morphogenesis (Figure 5a). Besides cardiac structure and function, ECM treatment downregulated ECM deposition genes, especially in 3‐month‐old iCMs. *COL3A1* was upregulated only in advanced age iCMs in an ECM dose‐dependent manner (Figure 5e). Regarding cellular stress, the genes we have dominantly seen in the advanced aged iCMs (*NPPA*, *NPPB*) were downregulated in all and were almost halved with the high concentration ECM treatment (Figure 5f).

At the protein level, ECM lowered the SASP components increased with cellular aging, such as the aging markers SERPINE1, IL‐8, IGFBP‐2, and VCAM (Figure 5g). ECM also decreased the maladaptive aging response associated cytokine, and chemokines (THBS1, CHI3L1, IL33) in 3‐month and 6‐month‐old iCMs. However, ECM treatment had a different effect on the advanced aged iCM than on 3‐month and 6‐month‐old iCMs (Figure 5g). Pro‐aging Ras and MAPK signaling pathway proteins (i.e., IL‐1b, FGF2) were highly expressed in advanced aged iCMs.

### 2.6 ECM treatment effect on aged iCM beating without any stress conditions

**FIGURE 6:**
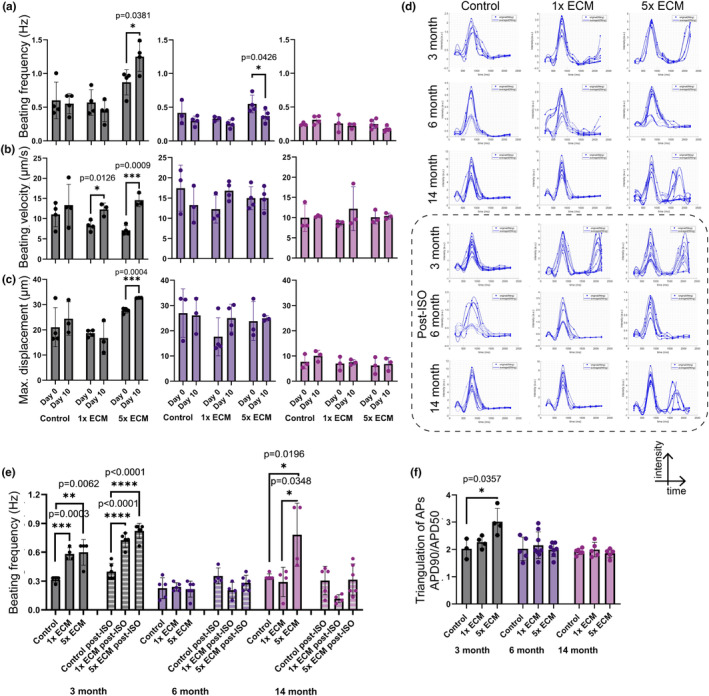
ECM treatment effect on aged iCM beating kinetics. (a) Beating frequency, (b) beating velocity of spontaneous cell beating and (c) maximum displacement of a pixel in a frame due to spontaneous beating before (Day 0) and after (Day 10) ECM treatment. (d) Overlapped action‐potential (AP) profiles of Ca‐transient from 10 or more beating readouts of aged iCMs before (top) and after isoproterenol treatment (post‐ISO, bottom). Graphs demonstrate iCM contraction as measured by recording the changes in the Fluo‐4 fluorescence intensity in time. Calculated (e) beating frequency, (f) time‐to‐peak and (g) triangulation of APs as the ratio of 90% to 50% decay from the calcium flux recordings. Statistical analysis was done using one‐way ANOVA with post hoc Tukey's test. ****p < 0.0001, ***p < 0.001, **p < 0.01, *p < 0.05, *n* ≥ 3. Data presented as mean ± standard deviation (SD).

We recorded the spontaneous beating of the aged iCMs on Day 10 of the ECM treatment. The initial beating frequencies were recorded as 0.72 ± 0.24 Hz for 3‐month, 0.48 ± 0.19 Hz for 6‐month and 0.25 ± 0.10 Hz for advanced aged iCMs, expectedly decreasing with increasing cell age. High‐dose ECM treatment significantly increased the beating frequency (Figure 6a), beating velocity (Figure 6b) as well as maximum displacement indicating the beating robustness of 3‐month‐old iCMs (Figure 6c). However, we observed minimal if not negative effects of ECM treatment on the 6‐month‐old and advanced age iCM beating properties (Figure 6a–c).

Relatedly, the calcium flux during spontaneous beating of iCMs was recorded (Figure 6d) and beating frequency, maximum contraction (time‐to‐peak, TTP), 50% decay (APD50), and 90% decay durations (APD90) were calculated (Figure S2c). ECM showed a clear concentration dependent effect on the shape of the calcium transients (Figure 6d). ECM treatment increased the beating frequency in 3‐month‐old iCMs while causing twitching of the advanced aged iCMs (Figure 6d,e) (Video S1 and S2). In addition, when treated with high dose ECM, 3‐month‐old iCMs exhibited uniform ventricular‐type action potential (AP) profile with a noticeable shoulder during repolarization. This was also confirmed by the significantly increased AP triangulation (Figure 6f). The 6‐month and advanced aged iCMs exhibited inconsistent AP peak intensity, shape and duration and it worsened with the ECM treatment. In addition, these cells responded minimally to the drug treatment.

Moreover, we detected higher mitochondrial membrane potential (Red/Green ratio), indicating increased ATP generation potential in 3‐month‐old iCMs after 10‐day ECM treatment (Figure S2d,e). Interestingly, the mitochondrial potential for both 6‐month and advanced aged iCMs significantly decreased after ECM treatment. Referring to the strong correlation between mitochondrial membrane potential and ROS production, we also measured the cellular ROS levels and observed only minimal changes with cell age or ECM treatment. Although ECM increased mitochondrial activity in 3‐month‐old iCMs, it did not lead to increased ROS production (Supp. Figure S2f).

## 3 DISCUSSION

In this study, we described the aging profiles of human LV and chronologically aged iCMs, and developed aged human heart models using aged iCMs to investigate the effect of cell age on young ECM treatment outcomes. We demonstrated that young ECM is an effective treatment for MI, as it promoted the survival of cells and facilitated their beating recovery, particularly in younger iCMs. However, young ECM had unexpected negative effects as a preventative therapy for advanced aged iCMs in the absence of any stress conditions. Despite its origin in young human LV, ECM increased aging‐factor expression and impaired beating of advanced aged iCMs, challenging its use for preventative purposes, especially for the elderly.

Many researchers have presented the human LV transcriptome in association with various diseases and conditions, yet we do not have a complete understanding of human heart aging. Age‐related changes reported for rodent hearts include myocyte hypertrophy, cardiac fibrosis, and reduced calcium transport across the sarcoplasmic reticulum membrane (S.‐K. Park & Prolla, 2005). Recent studies have identified up to seven cardiomyocyte states in human heart that the CMs in LV are marked with predominantly MYH6, ACTA1, and NPPA/NPPB. The later one being related to the disease state in some of the studies (Koenig et al., 2022). Although our analysis does not have the single‐cell resolution, we have successfully identified the related expressions and showed the age‐related changes between young and aged LVs, including impaired function. Here, we have confirmed the previously described age‐related pathophysiology at the transcriptomic level in human heart left ventricles (LV). We observed elevated levels of NPPA and NPPB in all aged LV samples (Figure 1), which is considered a hallmark of human aging and a protective hormonal response to mechanical stress to maintain cardiovascular homeostasis (Burnett et al., 2019). Additionally, DEGs in aged LVs showed an association with cGMP and HIF‐1 signaling pathways, as well as negative regulation of JUN kinase activity leading to hypertrophy and pronounced fibrosis in aged LVs (Windak et al., 2013). Complementing these results, we have detected increased collagen and collagen binding glycoproteins and decreased overall glycoproteome, which was recently associated with collagen fibrillation and age‐related cardiac functional decline, respectively (Franzka et al., 2021).

Prolonged culture (>1‐month) has typically been used as a CM maturation strategy (Hasan et al., 2020; Kamakura et al., 2013; Lewandowski et al., 2018; Vučković et al., 2022). Therefore, little was known about the capacity of aged iCMs to mimic human cardiac aging. Here, we report for the first time that functional (i.e., beating) 14‐month‐old advanced aged iCMs exhibit the hallmarks of cardiac aging at transcriptional, translational as well as cellular level, including adverse cardiac modeling, hypertrophy, and SASP (Figures 2 and 3). We observed reexpression of fetal genes (i.e., *TNNI1* and *MYH6*), induction of pre‐ANF (*NPPA*), pre‐BNP (*NPPB*), and *ACTA1* in the advanced aged iCMs similar to the distinct molecular phenotype associated with pressure overload‐induced hypertrophy. Additionally, advanced aged iCMs displayed beta‐adrenergic receptor expression levels that resemble those of 1‐month‐old iCMs (Figure 2d,e). The predominant expression of *ADRB2* (fetal‐type) and low expression of *ADRB1* (adult‐type) indicate β‐adrenergic desensitization can be seen during cardiac aging as well as in immature CMs. Although ADRB1 is also downregulated in ventricular CM pathogenic variants (Reichart et al., 2022), early studies suggest that the heart develops immature features with aging, including a dependence on transsarcolemmal calcium influx during contraction rather than calcium stored in the sarcoplasmic reticulum as in the adult heart (Bassani, 2006; Sawmiller et al., 1998). Here, we showed that 14‐month‐old advanced aged iCMs have demonstrated similarities with the ventricular CM clusters displaying high expression of sarcomere proteins (MYH7, MYL2), calcium‐mediated processes (RYR2, SLC8A1) and transcription factor that activates ventricular gene expression (IRX4) (Koenig et al., 2022; Litviňuková et al., 2020).The advanced aged iCMs displayed high stress‐related genes that are often associated with aging and disease states (Koenig et al., 2022) (Figure 2). Moreover, we verified that these cells were non‐proliferative cardiac cells with mature cardiac structural expression and aging hallmarks including age‐pigment accumulation, SASP expression, and functional impairment (Figure 3d).

We observed another phenomenon commonly seen in senescent cells. Despite high levels of initiator caspase CASP9, advanced aged iCMs had low levels of CASP3 (Figure 2b,h), indicating the central apoptotic machinery downstream CASP9 was inactivated and the upregulation of CASP9 was independent of apoptosis. Additionally, the increased resistance of advanced aged iCMs to apoptosis (Figure S1c) suggests a survival strategy specific to advanced aged iCMs, as also observed in aged olfactory bulb neurons but not in young counterparts (Ohsawa et al., 2009). This is known as the trade‐off between senescence and apoptosis (Hu et al., 2022), and the surprising results of ECM treatment in the absence of stress conditions might be due to this delicate balance. Using the heart tissue model with different aged iCMs, we demonstrated the critical role of ‘cell age’ in determining ECM treatment efficacy and outcome. Consistent with previous preclinical and Phase I clinical studies (Chen et al., 2016; Ozcebe et al., 2021; Singelyn et al., 2012; Traverse et al., 2019; Z. Wang et al., 2019), young ECM improved post‐MI beating recovery (Figure 4a–d). However, the effect of ECM on the post‐MI survival rate was highly cell age dependent, with only the younger cell group showing higher survival in response to ECM treatment (Figure 4e–h). Although young ECM enhanced oxidative stress coping mechanisms in advanced aged iCMs (Figure S2), a similar effect shown in a recent study revealing the ROS scavenger activity of ECM (R. M. Wang et al., 2022), this did not translate into an increase in their survival rate. The desensitization of advanced aged cells due to reduced cellular activity and function has long been known and also observed in the human heart as it ages (Ozcebe et al., 2021; Park, 2017). However, such a difference in previous studies has not been reported because samples are pooled together regardless of age to show the global effect of ECM therapies.

Current MI guidelines do not differentiate treatment based on age or sex, hence the treatment efficacies are suboptimal in the elderly and women. For this study, we acknowledge the potential sex‐based differences at both the gene and protein levels. A recent study on transcriptional diversity of the human heart (*n* = 7, ages: 39–60) reported 17 genes that exhibited sex‐based differential expression within cardiomyocytes (i.e., *NEB*, *PBX3*) (Tucker et al., 2020). Another study on sex‐related protein expressions in hypertrophic cardiomyopathy patients (*n* = 26, ages: 48.5 ± 17.7 (F) and 49.8 ± 15.5 (M)) reported 46 proteins that were differentially expressed in the female and male groups (i.e., tubulins and HSPs)(Schuldt et al., 2021). However, as we found no evidence for a sex‐based differential expression in the genes or proteins of interest (Figures 1, 2, 3), we did not separate our samples by sex.

Studies have demonstrated the great potential of young ECM therapies in promoting post‐MI recovery and regeneration, suggesting its use for also preventative purposes. We demonstrated similar transcriptomic and related translational changes reported for post‐MI ECM therapy results including reduced CM apoptosis, improved cardiac structure and function in 3‐month‐old iCMs treated with ECM (Figure 5). Related to the improved ion transport and calcium signaling expressions (Figure 5b–d), ECM increased beating frequency and robustness (Figure 6a–c), and 3‐month‐old iCMs displayed more mature ventricular‐type AP profiles (Figure 6d,f). In addition, ECM treatment improved structural and functional cardiac maturity and decreased SASP (i.e., SERPINE1, IGFBP2, and interleukins), ECM deposition, and stress‐related expressions in 3‐month‐old iCMs in a dose dependent manner (Figure 5b–f). We acknowledge the immature nature of iCMs, therefore observed maturation with the ECM treatment was expected. However, the ECM effect on SASP is noteworthy as SASP‐centered approaches are emerging as alternatives to target senescence‐associated diseases.

When we repeated the same ECM treatment for the 14‐month‐old advanced aged iCMs, we got unexpected results. The impact of “cell age” on the outcome of ECM treatment was particularly significant in the absence of stress conditions. In fact, advanced aged iCMs were minimally or negatively affected by the ECM treatment. ECM disturbed the calcium handling abilities of the advanced aged iCMs and caused irregular beating along with twitching (Figure 6d,e). In addition, ECM increased SASP, namely CXC chemokines and activated IL‐6/JAK–STAT pathway (Figure 5g) in advanced aged iCMs suggesting that young ECM exerted hypertrophic stress on advanced aged iCMs. As per our observations on the beating properties of advanced‐aged iCMs (Figure 6a), the elevation of pro‐inflammatory cytokines is often associated with impaired cardiac function (Jin et al., 2018). Moreover, a highly conserved pro‐aging RAS/MAPK signaling pathway was upregulated in advanced aged iCMs after ECM treatment in a dose‐dependent manner (Figure 5g).

Old age is associated with worse treatment outcomes and patient age is determinant in decision‐making and treatment selection in many disease conditions, including, breast cancer (Sio et al., 2014) and schizophrenia (Targum et al., 2017). This study highlights that age is also a critical determinant in the treatment of CVDs. Despite recent advances in ECM therapies, its efficacy and outcomes in elderly patients remain limited by the lack of data. Our results clearly demonstrated that the advanced aged iCMs, representing the elderly, did not benefit equally from the post‐MI young ECM treatment as the younger iCMs, and were even adversely affected in the absence of stress conditions. On that note, we acknowledge that culturing cells for extended periods of time is not convenient regarding time and cost. As an alternative, DNA‐damaging agents, cardiotoxic drugs (i.e., doxorubicin) and oxidants (i.e., H_2_O_2_) can be used to induce age‐independent premature senescence (SIPS), and mimic cardiac aging (Lazzarini et al., 2022). The use of SISP cells could offer an alternative in situations where invasive (i.e., patch clamp) or reseeding requiring (i.e., microelectrode array, MEA) qualitative methods are not suitable for advanced aged iCMs. Regardless, age‐appropriate cardiac models, such as the one presented here, are needed in the cardiac tissue engineering field to facilitate CVD therapy studies and enhance our understanding of cardiac aging.

## 4 CONCLUSION

Our study revealed age‐dependent transcriptional alterations in nonfailing human heart LVs, with a sole focus on aging without any coexisting disease states. Moreover, we showed that chronologically aged iCMs are excellent candidates to mimic aged heart behavior, and aged heart models using age‐appropriate iCMs are valuable for studying age‐dependent efficacy and outcome of the CVD therapies. Our results demonstrated that the ECM response is highly dependent on cell age and stress conditions. Therefore, there is a need for age‐appropriate cardiac models in translational research to develop personalized treatments for the elderly population, and to move beyond the “one‐size‐fits‐all” approach in ECM therapies.

## 5 MATERIALS AND METHODS

### 5.1 Donor heart harvest

De‐identified human hearts that were deemed unsuitable for transplantation and donated to research, were acquired from Indiana Donor Network under the Institutional Review Board (IRB) approval for deceased donor tissue recovery. Human heart tissues were grouped as young (from <30 years‐old patients, *n* = 3), and aged (from 50 < years‐old patients, *n* = 3). For storage, hearts were dissected into its chambers and kept separately in a − 80°C freezer until use. We only used the young left ventricles (*n* = 3) for the ECM treatments.

### 5.2 Decellularization of human heart tissue for matrix preparation

Left ventricles from young donors were sectioned and decellularized following previous decellularization protocol (Basara et al., 2021). Briefly, we first stripped the fatty tissue around the left ventricular myocardial tissue and sliced the tissues in thin sections (<1 mm). To decellularize, tissues were washed in 1% (wt/vol) sodium dodecyl sulfate (SDS) (VWR, #97062) for 24 h or until white transparent tissue was obtained, then in 1% (wt/vol) Triton 100‐X (Sigma‐Aldrich, #A16046) for 30 min. After decellularization, samples were washed thoroughly with DI water to remove any residual detergent. To delipidize, tissues were washed with the isopropanol (IPA) for 3 h then rehydrated in DI and treated with 50 U/mL DNase (Millipore Sigma, #10104159001) for 8 h followed by an overnight DI rinse. All steps were conducted with constant agitation at RT.

Prepared ECMs were lyophilized and pulverized with liquid nitrogen. ECM powder was digested in a 1 mg/mL pepsin (Sigma‐Aldrich, #P6887) in 0.1 M HCl (10:1, w/w, dry ECM:pepsin) at RT with constant stirring until a homogeneous solution was obtained. The insoluble remnants were removed by centrifugation, the supernatant was neutralized using 1 M NaOH solution, and used immediately to prevent degradation. Prior to experiments, we measured the total protein concentrations using Rapid Gold BCA Assay (Thermo Scientific, # A53227) and diluted ECM solutions to either 0.01 mg/mL (1x) or 0.05 mg/mL (5x) with the culture media.

### 5.3 Human iPSC cell line

The cell line used in this study is DiPS 1016 SevA (RRID: CVCL_UK18) from human dermal fibroblasts obtained from Harvard Stem Cell Institute iPS Core Facility. Cells were cultured in humidified incubators at 37 °C and 5% CO2. Human iPS cells were cultured routinely in mTeSR‐1 media (StemCell Technologies, #05825) on 1% Geltrex‐coated plates (Invitrogen, #A1413201). At 80–85% confluency, cells were passaged using Accutase (StemCell Technologies, #07920) and seeded at 1.5 × 10^5^ cells/cm^2^ on well plates with Y‐27632 (ROCK inhibitor, 5 *μ*M), (StemCell Technologies, #129830–38‐2) in mTeSR‐1 media. The culture was maintained with daily media changes until 90% confluency was reached.

### 5.4 Culturing iPSC‐derived cardiomyocytes

Once 90% confluency was reached, cardiac differentiation was initiated following canonical Wnt pathway (Ozcebe et al., 2021). To direct cardiac differentiation, cells are sequentially treated with CHIR99021 (12 *μ*M) (Stemcell Technologies, #72052) for 24 h followed by RPMI 1640 medium with B‐27 supplement without insulin (2%) (Gibco, #A1895601) (CM(−)). Cells were then treated with Wnt pathway inhibitor IWP‐4 (5 μM) (Stemcell Technologies, #72552) for 48 h followed by CM(−) for 48 h. From Day 9 on, cells were maintained in RPMI 1640 medium with B‐27 (2%) (Gibco, #17504044) (CM(+)) and media was changed every 3 days. To improve the CM purity, cells metabolically selected by culturing in glucose‐depleted medium supplemented with fatty acid (1× Linoleic Acid‐Oleic Acid‐Albumin (Sigma, L9655)) for 5 days. Purified iCMs were cultured for 3 months, 5–6 months, and 13–14 months.

### 5.5 ECM treatment experiments

Myocardial infarction (MI) experiment was mimicked in two parts as ischemic phase (I) and reperfusion injury (RI). Aged cells were incubated under anoxic conditions (37 °C, 5% CO_2_, 0.1% O_2_) for 3 h (I), then moved to normoxic conditions (21% O_2_) for 12 h (RI). During ischemia, cells were incubated in anoxia‐equilibrated RPMI 1640 medium without glucose (Corning, #10043CV) with B‐27 supplement without antioxidants (2%) (Gibco, #10889038). During RI, cells were incubated with CM(+) medium alone or supplemented with decellularized ECM (1x or 5x concentration). For functional recovery, spontaneous beatings were recorded at 1 h, 3 h, 6 h, and 12 h RI. At 3 h RI, cellular ROS was measured and at 12 h RI apoptosis‐related proteome was profiled (R&D Systems, #ARY009), and survival rate was measured via live/dead staining (Abcam, #ab115347).

Aged cells were treated with decellularized ECM for 10 days and control groups were maintained in CM(+) media throughout the experiment. Cells were screened for their relative cytokine content and gene expressions before and after ECM treatment. After treatment, spontaneous beating of the cells as well as mitochondrial health (ThermoFisher, MitoProbe JC‐1, #M34152) and cellular ROS (Abcam, Cellular ROS Assay #ab186029) were assessed.

### 5.6 RNA isolation

Cells were rinsed with PBS, collected with trypsin, and stored in a − 80°C freezer for future RNA isolation. For RNA isolation, frozen cells were thawed and centrifuged to remove the freezing media. The pellet was then processed following the RNeasy Mini Kit (Qiagen, #74104) protocol. Briefly, cells were disrupted using the lysis buffer and the same volume ethanol added to the lysate. The sample is then applied to the RNeasy mini spin column, collected on the membrane, and finally RNA was eluted in RNAse‐free water. RNA purity was confirmed, concentration was measured using a Nanodrop 2000 spectrophotometer, and samples were sent to the core facility at Ohio State University.

### 5.7 Gene expression analysis

mRNA levels were quantified using NanoString Technology. An nCounter custom codeset was designed for the identification of genes of interest related to iCM maturity, function and apoptosis with a total of 64 genes including 5 housekeeping genes (*B2M*, *EEF1A1*, *GAPDH*, *RNPS1*, and *SRP14*), selected based on a publication (Murphy et al., 2019). RNA inputs of 100 ng were used for hybridization and placed on a cartridge for the NanoString reader. The output files (RCC files) were loaded into nSolver Analysis software. Data were run for quality control and background normalization, then genes of interest were normalized to the housekeeping genes. For visualization purposes, heatmap of log_2_FC for the differentially expressed genes was generated using the nSolver analysis software v4.0. Complete linkage hierarchical clustering method with Euclidean distance was used to cluster the human tissues and iCMs.

Gene Ontology (GO) Analysis was performed on the obtained relative expression data. Comprehensive analysis was performed using an online database via EnrichR for biological processes and enrichment analysis. Data were extracted from the output dataset and graphed manually using the *p*‐value provided. Proteomic interactions of the same relative expression data were also classified through KEGG‐based proteomapping software and are presented as obtained.

### 5.8 Proteome analysis

Mass spectrometry (MS) was used to determine the protein composition of the human heart tissues (*n* = 3 for each age group, three technical replicates). The samples were digested in the digest solution (5 M urea, 2 M thiourea, 100 mM ammonium bicarbonate and 50 mM dithiothreitol, pH 8.0) at 4°C for 24 h with constant stirring. The soluble ECM protein concentration was measured with the Pierce Rapid Gold BCA Assay (Thermo Scientific). Samples were further processed using S‐TrapTM mini spin column digestion protocol and eluted peptides were used for MS analysis. The ECM components for human LV were identified and quantified using Thermo Scientific Proteome Discoverer and PEAKSOnline Proteomics Server software.

The relative cytokine content of the aged iCMs before and after ECM treatment and of the human heart ECM were obtained using the Human XL Cytokine Array Kit (ARY022B, R&D Systems). Briefly, cell lysates were obtained by disrupting the cells using the lysis buffer supplemented with protease inhibitor cocktail and tissue extracts were obtained by 1% Triton‐X incubation. The relative expression levels of apoptosis‐related proteins of post I/RI iCMs (*n* = 3) were analyzed using the Proteome Profiler Human Apoptosis Array (R&D Systems, #ARY009), according to the manufacturer's instructions.

For all, protein concentrations were normalized before starting the arrays and incubated overnight with pre‐blocked membranes at 4°C. At the end, the unbound proteins were rinsed away, the membranes were incubated with the streptavidin‐HRP, then developed using chemiluminescent detection reagent mixture. For quantification, the background was removed, and the pixel density of each spot was measured using ImageJ.

### 5.9 Beating analysis

Block‐matching algorithm was performed using a custom MATLAB code. Briefly, the spontaneous beating of the iCMs were recorded as bright field videos (*n* = 3–4 ROI per sample) and exported as a series of single‐frame images files. The images were then divided into square blocks of NxN pixels. Movement of a given block at the kth frame is tracked by matching its intensity to the (k + 1)th frame within a square window of a fixed width flanking from each side. The pixel values were determined based on the speed of calculation and accuracy of the block‐matching method. The matching criterion used for block movement is Mean Absolute Difference as described previously (Acun et al., 2019).

### 5.10 Calcium staining

The calcium (Ca^2+^) transient of iCMs was assessed by incubating cells in Ca2 + −sensitive Fluo‐4 AM (Life Technologies) solution, as instructed by the manufacturer. Cell beating was recorded in real‐time using a fluorescence microscope (Axio Observer.Z1, Zeiss, Hamatsu C11440 digital camera) at 30 ms exposure for 30 s. To assess the drug response, we treated the cells with 1 μM isoproterenol (ISO) for 10 min at 37°C. Spontaneous beating before and after ISO was recorded. The baseline for calcium transient and an initial beat rate of the cells were obtained from pre‐drug recordings (5 images/well, and 3 wells/age group). Rate of Ca^2+^ release (time to peak Ca^2+^ transient amplitude) and action potential duration at 50% (APD50) and 90% (APD90) of the amplitude were calculated from the raw data obtained from intensity versus time plots. Triangulation of the APs were calculated as the ratio of APD90 to APD50, as an additional indication of the AP profiles.

### 5.11 Immunostaining

Cells were fixed with paraformaldehyde (4% PFA) for 15 min at RT and treated with the permeabilization solution (0.1% Triton‐X 100) for 20 min at RT, followed by the blocking solution (10% Goat serum) for 1 h at RT. Cells were incubated with the primary antibodies for TNNT2 (Abcam, ab45932), Ki67 (Thermo Fisher, 14–5698‐82), p21 (Abcam, ab54562) (all diluted at 1:150 in goat serum) overnight at 4°C and with the species‐appropriate secondary antibodies (Life Technologies) (dilutions: 1:300) for 6 h at 4°C. Images were captured using Zeiss LSM 900 with Airscan 2 confocal microscope and post imaging processing was performed using Zeiss Zen software and ImageJ.

### 5.12 Statistical analysis

The mean ± standard deviation (SD) was reported for all replicates. One‐way ANOVA with post hoc Tukey's test was used to assess the statistically significant differences using GraphPad Prism version 8. All *p* values reported were two‐tailed, and *p* < 0.05 was considered statistically significant. Sample size (*n*) ≥ 3 for individual experiments.

## AUTHOR CONTRIBUTIONS

S.G.O. and P.Z. designed research, S.G.O. performed research, analyzed data, S.G.O. and P.Z. conducted review and editing, P.Z. provided funding, project administration, and resources, S.G.O. wrote the paper and P.Z. revised the paper.

## FUNDING INFORMATION

This work was supported by the National Science Foundation CBET grant number [1651385] and [NSF‐1805157].

## CONFLICT OF INTEREST STATEMENT

The authors have no competing interest to disclose.

## Supporting information


Figure S1.

Figure S2.

Video S1.

Video S2.

## Data Availability

The raw/processed data required to reproduce these findings can be shared upon reasonable request.
