# Supplementary figures and images for: In need of age‐appropriate cardiac models: Impact of cell age on extracellular matrix therapy outcomes
Source: Aging Cell. 2023 Oct 6;22(11):e13966. doi: 10.1111/acel.13966 (PMC10652343; doi:10.1111/acel.13966)

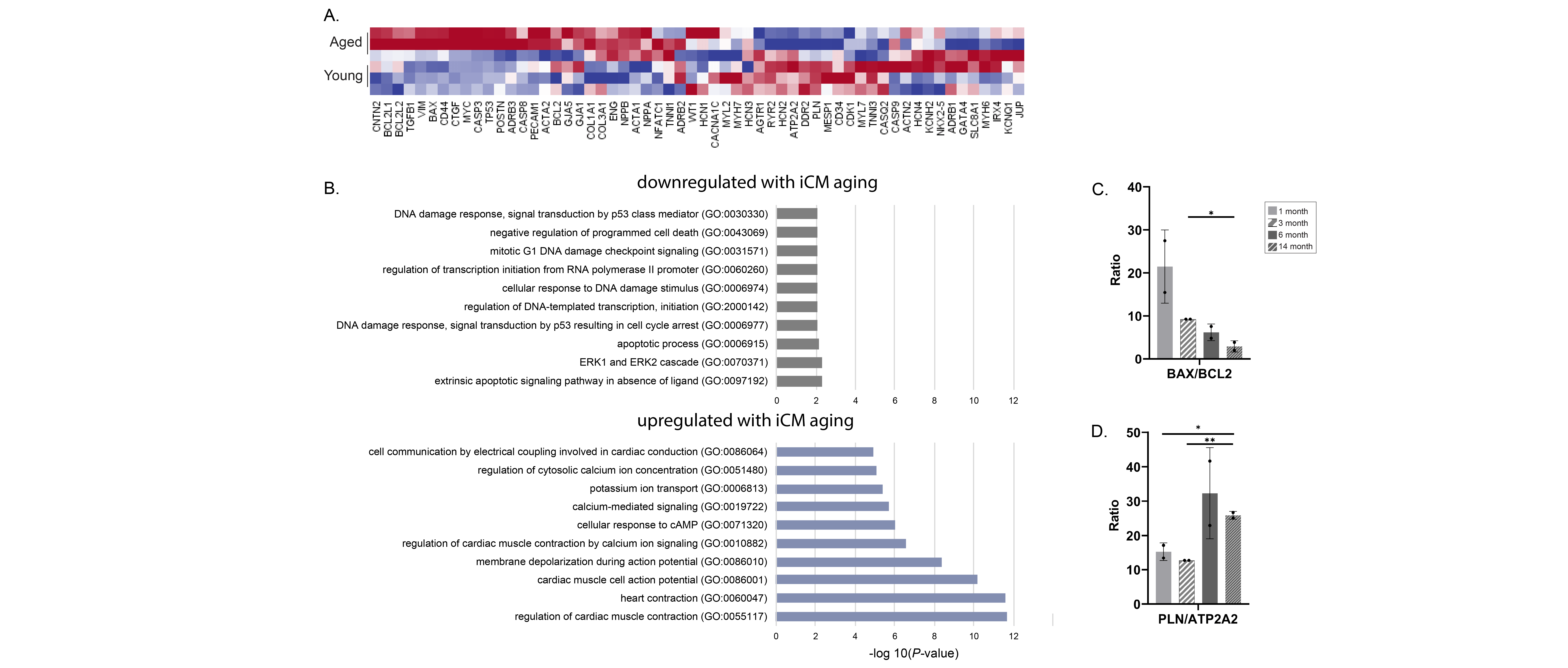

Supplement: Supplementary file 1 — Figure S1. [file ACEL-22-e13966-s004.png]

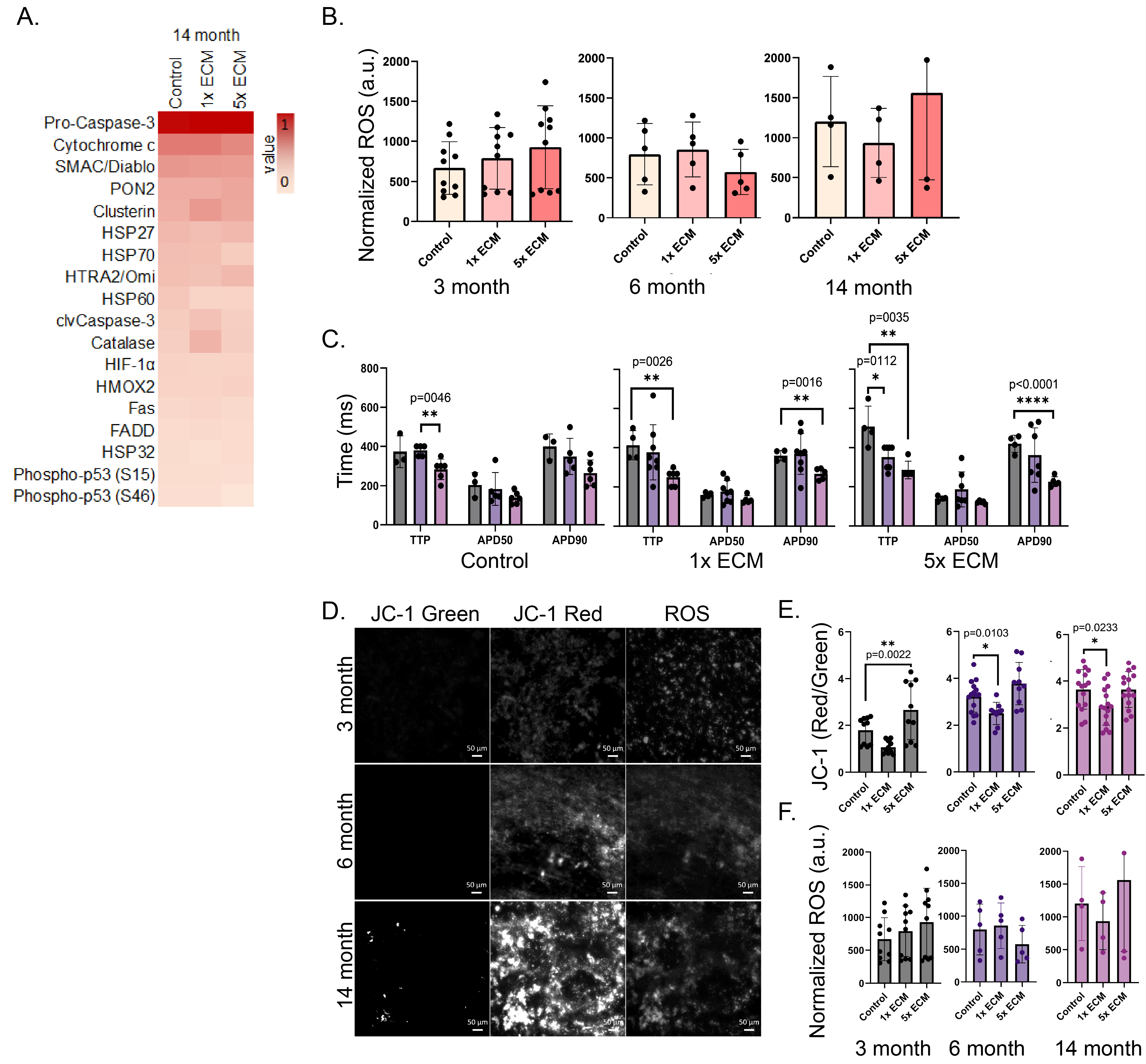

Supplement: Supplementary file 2 — Figure S2. [file ACEL-22-e13966-s001.png]
